# Postoperative Intra-Pouch Mucosal Bridge Formation in a Child with Ulcerative Colitis

**DOI:** 10.70352/scrj.cr.25-0045

**Published:** 2025-05-02

**Authors:** Yuhki Koike, Koki Higashi, Yuki Sato, Shinji Yamashita, Yuka Nagano, Tadanobu Shimura, Takahito Kitajima, Kohei Matsushita, Yoshinaga Okugawa, Yoshiki Okita, Yuji Toiyama

**Affiliations:** 1 Department of Gastrointestinal and Pediatric Surgery, Mie University Graduate School of Medicine, Tsu, Mie, Japan; 2 Department of Cancer Genome, Mie University Graduate School of Medicine, Tsu, Mie, Japan

**Keywords:** children, mucosal bridge, ulcerative colitis

## Abstract

**INTRODUCTION:**

Restorative proctocolectomy with construction of an ileal J-pouch anal anastomosis is an established gold standard procedure for managing ulcerative colitis. One of the reported complications is a residual mucosal bridge as a result of leaving an apical bridge remnant when constructing the ileal J-pouch. However, now that the surgical procedure is well established, such complications rarely occur.

**CASE PRESENTATION:**

A 12-year-old girl presented to our hospital because of anal pain. She had undergone three-stage surgery for ulcerative colitis refractory to medical therapy, the third stage (stoma closure) having been performed 1 month before the present admission. A computed tomography scan to investigate the possibility of a perianal or pelvic abscess showed no abscess, but revealed what appeared to be a thickening of the wall of the J-pouch, suggestive of pouchitis. Endoscopy revealed a mucosal bridge crossing the anterior and posterior walls of the J-pouch, with a stapler line near the posterior wall's root; however, there was no evidence of pouchitis. While creating the J-pouch (during the second stage of surgery for ulcerative colitis), we had ensured that an apical bridge was eliminated with a linear stapler. Moreover, a contrast enema of the J-pouch during the present admission demonstrated interruption of contrast in the J-pouch. These findings led us to conclude that the mucosal bridge had probably formed postoperatively, after J-pouch creation. The patient underwent endoscopic resection of the mucosal bridge in the J-pouch using an XXS wound retractor transanally. Both ends of the bridge were cut three times with a 5-mm stapler and the bridge was resected. The patient was discharged after surgery, having experienced immediate resolution of anal pain and no complications. Pathological examination of the resected specimen showed that the ileal wall had bent toward the J-pouch lumen with fibrous adherence on the serosal side, indicating that the mucosal bridge had developed unintentionally post-stoma closure. Preoperative computed tomography showed limited pouch expansion, whereas postoperative computed tomography showed sufficient expansion.

**CONCLUSION:**

If anal pain develops following radical ulcerative colitis surgery (after ileal stoma closure), postoperative mucosal bridge formation should be included in the differential diagnosis.

## INTRODUCTION

Since Utsunomiya et al. reported restorative proctocolectomy with construction of an ileal J-pouch anal anastomosis (IPAA) for ulcerative colitis (UC), this procedure has become the gold standard worldwide.^1)^ However, when it was first developed, postoperative defecation disfunction and anorectal pain sometimes occurred, one of the causes being a residual apical bridge at the time of J-pouch creation.^2)^ Kusunoki et al. reported that a residual apical bridge caused difficulty with evacuation and perianal pain and named this as apical bridge syndrome.^3)^ Thereafter, postoperative apical bridge syndrome rarely occurred because surgeons took care to ensure separation of the apical bridge at the time of J-pouch creation. In this report, we describe an extremely rare case in which a mucosal bridge resembling an apical bridge had formed after stoma closure in a patient in whom the apical bridge had been securely dissected at the time of J-pouch creation.

## CASE PRESENTATION

**Fig. 1:**
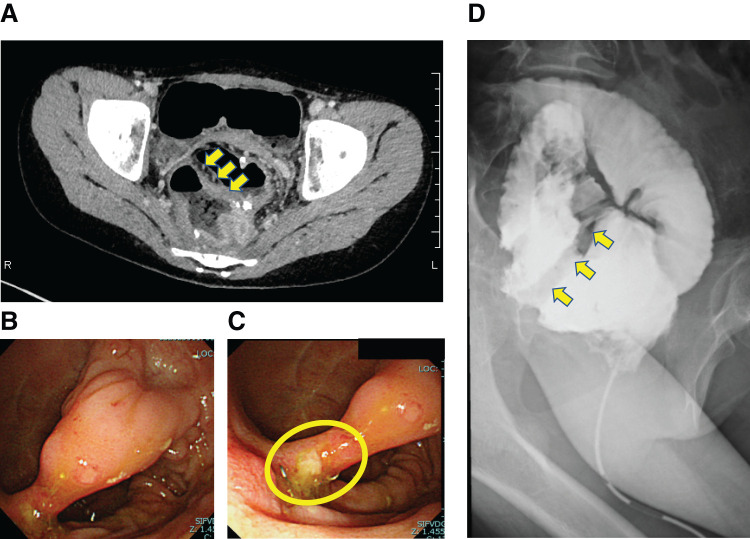
Preoperative image examination findings. (**A**) CT on admission showing what was interpreted as thickening of the wall of the ileal J-pouch. Retrospectively, this was recognized as a mucosal bridge with a stapler line at one end (yellow arrows). (**B**) Full endoscopic view of a mucosal bridge in the J-pouch. (**C**) Root of mucosal bridge. A stapler line can be identified at the root of the mucosal bridge (yellow circle). (**D**) Contrast enema image on admission. Interruption of contrast is apparent in the J-pouch (yellow arrows).

**Fig. 2:**
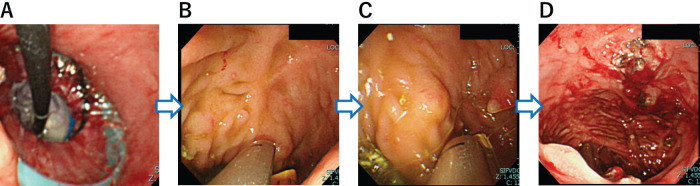
Endoscopic images during surgery. (**A**) An XXS wound retractor has been placed transanally to enable visualization of the J-pouch. (**B**) One side of the mucosal bridge has been severed using a 5-mm linear stapler. (**C**) The other side of the mucosal bridge has also been severed using a 5-mm linear stapler twice. (**D**) Endoscopic image after mucosal bridge resection.

**Fig. 3:**
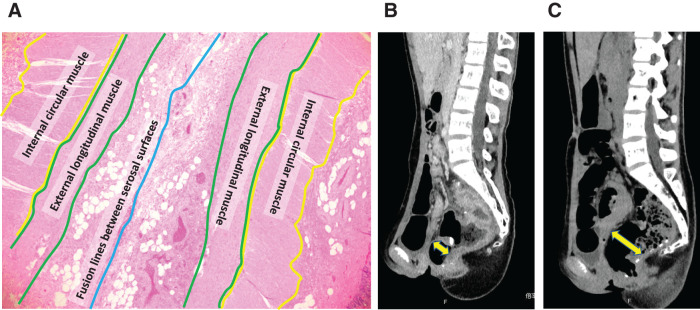
Pathological image and comparison of pre- and postoperative CT. (**A**) Photomicrograph of the excised mucosal bridge. The ileal wall has bent toward the J-pouch lumen with fibrous adherence on the serosal side. (**B**) CT on admission (sagittal section). A mucosal bridge is restricting expansion of the J-pouch. (**C**) Postoperative CT (sagittal section). Resection of the mucosal bridge has achieved expansion of the J-pouch.

A 12-year-old girl presented to our hospital with anal pain. The patient was diagnosed with UC 3 years prior and achieved remission through induction therapy with steroids, followed by maintenance with 5-aminosalicylic acid and azathioprine. However, a relapse occurred 3 years after the initial onset, and treatments with anti-TNF-α antibody agents proved ineffective. Consequently, she opted for a three-stage surgical approach to manage her condition. One month prior to admission, she underwent ileal stoma closure as the final stage of this staged operation. Upon admission, her vital signs were as follows: body temperature of 37.3°C, blood pressure of 105/76 mmHg, and pulse rate of 106 bpm. Physical examination revealed mild tenderness in the lower abdomen. Digital anorectal examination showed no evidence of anastomotic stenosis; however, the patient reported perianal pain. On admission, laboratory findings revealed a leukocyte count of 3510/μL, with neutrophils comprising 71%. The C-reactive protein level was 6.82 mg/L, and the erythrocyte sedimentation rate was 24 mm/h, consistent with an elevated inflammatory response. Following stoma closure, the patient developed anorectal pain and lower abdominal pain, accompanied by signs of an elevated inflammatory response. These findings raised suspicion of a postoperative perianal abscess or pouchitis. Consequently, a CT scan was performed upon admission for further evaluation. The CT revealed no evidence of a perianal abscess, however, thickening of the wall of the J-pouch was detected, suggesting pouchitis, so an endoscopy was performed (**Fig. 1A**). Pouchoscopy revealed a mucosal bridge crossing the anterior and posterior walls of the J-pouch. A stapler line was identified in close proximity to the root of the posterior wall; however, there was no evidence of pouchitis (**Fig. 1B**, **1C**). At the time of J-pouch creation, we had confirmed that an apical bridge had been dissected by a linear stapler. Furthermore, the contrast enema performed to visualize the J-pouch immediately after the current admission revealed an interruption in the contrast medium within the J-pouch (**Fig. 1D**). We concluded on the basis of these findings that the mucosal bridge had developed as a result of formation of adhesions sometime between the creation of the J-pouch and stoma closure. We resected endoscopic mucosal bridging in the J-pouch. Under general anesthesia, an XXS wound retractor was placed via a transanal approach (**Fig. 2A**). A 5-mm endoscope was inserted directly from the wound retractor and adjusted so that the entire mucosal bridge was in view. A 5-mm linear stapler (JustRight 5mm Stapler; AMCO Inc., Tokyo, Japan) was inserted through the retractor (**Fig. 2B**, **2C**), and the root of the mucosal bridge was completely resected once on one side and twice on the other side, using the endoscope while viewing the area where the stapler was fired (**Fig. 2D**). The operative time was 79 minutes, with a minimal estimated blood loss of 2 mL. The patient was discharged from the hospital, her preoperative anal pain having been relieved immediately by this surgical procedure. Histopathological examination of the resected specimen revealed that the ileum wall was bent convexly toward the lumen of the J-pouch and the bridge had adhered to its serosal side with fibrous tissue, suggesting that the mucosal bridge had formed postoperatively (**Fig. 3A**). A preoperative CT had shown that a mucosal bridge was limiting the expansion of the pouch (**Fig. 3B**), whereas a postoperative CT showed that the pouch had expanded sufficiently (**Fig. 3C**).

## DISCUSSION

As formation of a J-pouch and IPAA became standard procedures, the techniques for performing them improved and it became widely known that the crucial component of this procedure is effective intraoperative separation of an adequate apical bridge. Sakanoue et al. were the first to report a technique for guiding the apical bridge out of the anus using a Nelatone catheter and dissecting both ends with a linear stapler to separate an apical bridge after IPAA surgery.^2)^ However, even after the year 2000, there were still reports of apical bridge syndrome after radical UC surgery, and various techniques were reported for managing apical bridges. For example, Takagi et al. reported a procedure in which an endoscope is inserted transanally to create a J-UP shape and the apical bridge in the J-pouch visualized, after which a transanally inserted linear stapler is used to dissect the apical bridge.^4)^ By contrast, Poola et al. reported a procedure in which an endoscope is inserted through the ileal stoma and guided into the J-pouch and the apical bridge visualized in the field of view, after which a transanally inserted linear stapler is used for dissection.^5)^ Subsequently, with the spread of the perfected IPAA technique (intraoperative dissection of an apical bridge) worldwide, there have been no more reports concerning the management of postoperative apical bridges since 2009.

In the case we experienced in this study, an apical bridge was definitely eliminated by using a linear stapler at the time of J-pouch creation. However, a contrast enema after the patient was readmitted with complaints of anal pain revealed an interruption in the contrast medium within the J-pouch. Moreover, as shown in **Fig. 1A** and **3B** on CT and **Fig. 1B** and **1C** on endoscopy, a mucosal bridge crossed the anterior and posterior walls of the J-pouch, a stapler line being identified at the root of one of the ends of the bridge. Furthermore, histopathological examination revealed that the ileal wall in the J-pouch protruded into the lumen and the serosal surfaces had adhered to each other. The reformation of the postoperative mucosal bridge in this case is likely attributable to the adjacent stapler lines within the ileal pouch, although this remains speculative. Notably, such an occurrence has not been documented in adult patients. In pediatric patients with a narrow pelvis, as seen in this case, it is possible that even when the apical bridge is successfully separated during surgery, the postoperative proximity of the stapler lines in the pouch can contribute to the re-formation of the mucosal bridge. Despite careful intraoperative separation, the stapler lines remained closely situated within the pouch following surgery, leading to the observed recurrence of the mucosal bridge. This unique finding highlights the anatomical challenges in pediatric patients and the potential influence of stapler line proximity in postoperative outcomes. Based on these findings, we concluded that the mucosal bridge had most likely formed by postoperative adhesion of a portion of the stapler line in the J-pouch to the contralateral side of the ileum.

This is the first case report of such a mucosal bridge in the ileal pouch after UC surgery, including an adult case, except for a mucosal bridge caused by apical bridge syndrome, although this is a mucosal bridge seen in the transition of surgical techniques. While mucosal bridges may occasionally develop in the intestinal tract during the clinical course of inflammatory bowel diseases such as Crohn’s disease and UC, their formation is typically associated with ulcerative lesions in the intestinal wall. Notably, such bridges do not form in the absence of inflammation or ulcerative changes in the surrounding intestinal mucosa, as seen in this case. Furthermore, the mucosal bridge described here exhibited stapler lines at its tips—a feature never observed in mucosal bridges caused by ulcerative lesion of inflammatory bowel disease. These findings highlight the extraordinary rarity of a mucosal bridge featuring stapler lines and arising in the absence of any surrounding mucosal ulceration, as observed in this postoperative ileal pouch case.

There have been several reports on how to manage post-radical surgery apical bridges, as described above. In this case, the mucosal bridge was confined to the ileal pouch, and during the initial endoscopic examination, it was evident that visualizing the entirety of the mucosal bridge would be challenging when the stapler firing had been performed laparoscopically. Additionally, despite the absence of anorectal stenosis, we opted against performing the procedure under direct vision. This decision was made out of concern that opening the anus under direct vision in the narrow pelvis of a young girl during the early postoperative period could result in anal fissures and potential damage to the ileal J-pouch. Considering the above-mentioned factors during preoperative surgical planning, in the present case, a 5-mm stapler was used endoscopically via a transanal approach. This was not only a simple procedure, even for a pediatric patient with a small anus, but also achieved rapid postoperative resolution of anal pain with no complications, suggesting that it is a safe and effective procedure.

Our patient’s perianal pain was exacerbated by straining. Pre- and postoperative CT scans comparison showed that pouch expansion had been achieved. Furthermore, her perianal pain resolved promptly after surgery, suggesting that exacerbation of anal pain upon straining may be a characteristic finding when an apical bridge develops postoperatively in a J-pouch (**Fig. 3B**, **3C**).

## CONCLUSIONS

We here present a rare case of mucosal bridging in the J-pouch of a pediatric patient who had undergone UC surgery and whose apical bridge had been securely eliminated at the time of creation of her J-pouch. When anal pain develops after radical UC surgery, mucosal bridging should be included in the differential diagnosis.

## ACKNOWLEDGMENTS

The authors thank Dr. Trish Reynolds, MBBS, FRACP, from Edanz (https://jp.edanz.com/ac) for editing a draft of this manuscript.

## DECLARATIONS

### Funding

All authors declare that they have received no funding support for this article.

### Authors’ contributions

Conception or design of the work: YK, YO, and YT.

Patient’s surgery and postoperative management: YK, KM, YN, YS, KH, and YO.

Data collection: YK, TS, and TK.

Interpretation of data for the work: YK.

Writing: YK.

All authors have read and approved the manuscript.

### Availability of data and materials

The dataset supporting the conclusions of this article is included within the article.

### Ethics approval and consent to participate

This work does not require ethical considerations or approval. Informed consent to participate in this study was obtained from the patient’s parents.

### Consent for publication

Written consent for publication was obtained from the patient’s parents, and informed consent for the study was also secured.

### Competing interests

All authors declare no competing interests regarding this article.

## References

[1] Utsunomiya J Iwama T Imajo M Total colectomy, mucosal proctectomy, and ileoanal anastomosis. Dis Colon Rectum 1980; 23: 459–66. 6777128 10.1007/BF02987076

[2] Sakanoue Y Shoji Y Kusunoki M Transanal division of an apical pouch bridge after restorative proctocolectomy with a J shaped reservoir. Br J Surg 1993; 80: 248. 8443672 10.1002/bjs.1800800245

[3] Kusunoki M Okamoto T Ikeuchi H Apical pouch bridge syndrome after ileal J pouch-anal anastomosis. Dig Surg 1996; 13: 474–7.

[4] Takagi K Nagata H Ishizuka M Endoscopy-assisted transanal division of an apical pouch bridge after restorative proctocolectomy with a J-shaped ileal pouch. Surg Laparosc Endosc Percutan Tech 2008; 18: 486–8. 18936672 10.1097/SLE.0b013e318180812a

[5] Poola VP Holubar SD Dozois EJ. Endoscopically assisted transanal division of pouch septum after ileal pouch-anal anastomosis for ulcerative colitis. Tech Coloproctol 2009; 13: 183. 19484340 10.1007/s10151-009-0478-2

